# Are Corneal Patients Accepting the Transplantation? The Cases of University of Gondar, Tertiary Eye Care and Training Center, Ethiopia

**DOI:** 10.1155/2019/4560649

**Published:** 2019-12-13

**Authors:** Yordanos Tsehai Jemberu, Yared Assefa Woldie, Wossen Mulugeta, Destaye Shiferaw Alemu, Getasew Mulat Bantie

**Affiliations:** ^1^Department of Ophthalmology, College of Medicine and Health Sciences, University of Gondar, Gondar, Ethiopia; ^2^Department of Optometry, College of Medicine and Health Sciences, University of Gondar, Gondar, Ethiopia; ^3^Department of Public Health, GAMBY Medical and Business College, Bahir Dar, Ethiopia

## Abstract

**Background:**

For so long, corneal diseases have been known as one of the leading causes to blindness in the global. This blindness might be due to failure to accept the corneal transplantation. Therefore, this study aimed to determine the prevalence and the root challenges for corneal transplantation acceptance at the University of Gondar tertiary eye care and training center, Ethiopia.

**Methods:**

An institution-based cross-sectional study was conducted among patients who had an indication for corneal transplantation at the University of Gondar tertiary eye care and training center since January 1, 2017, up to October 30, 2018. A structured questionnaire was used to collect the data and entered into Epi-Info 7 software and analyzed using SPSS version 20. Simple logistic regression was used to identify the associated factors of corneal transplantation acceptance. Associations between outcome and exposure variables were expressed by the adjusted odds ratio with a 95% confidence interval and *p* value <0.05.

**Result:**

A total of 116 patients with a mean age of 51 (±21) years participated in the study. The overall acceptance level of corneal transplantation was only 38.8% (95% CI: 29.93, 47.66). Patients with poor knowledge [AOR = 2.41; 95% CI: 1.90, 6.48] and an unfavorable attitude [AOR = 6.33; 95% CI: 2.42, 16.54] were significantly associated with the acceptance of corneal transplantation.

**Conclusion:**

The study revealed that the corneal transplantation acceptance level was very low. Hence, the government and other concerned stakeholders should give due emphasis to the awareness creation and behavior change communication strategies to increase the acceptance level of corneal transplantation.

## 1. Introduction

The corneal disease ranks as the fifth top leading cause of blindness in the world [1] and the second most prevalent ocular condition in developing countries [2]. The developing world shoulders 90% of the world's blindness [3], in which 80% of causative agents are being preventable or treatable [3]. When preventive strategies have failed, corneal transplantation is the most effective treatment to restore visual function for advanced corneal disease [3].

“Organ and tissue transplantation is a complex process with many legal, ethical, religious, and cultural barriers.” However, the cornea storage and transplantation are easier than other tissue and organs, making the corneal transplantation to be considered the world's most frequent type of transplantation [4].

Corneal transplants have been successfully performed on human subjects for over 100 years [5] and are a widely practiced surgical solution for blinding corneal opacity. The surgical techniques continue to evolve and increasingly are being used for indications that used to be treated with penetrating keratoplasty [6].

Although corneal transplantation is readily available in the USA and certain other regions of the developed world, the need for human donor corneas worldwide far exceeds supply [7]. For the past 15 years, from 2003 to 2017, the Eye Bank of Ethiopia (EBE) has extensively worked to overturn the burden of the country's corneal blindness. As a result, during this period, 1,818 corneas were collected, of which 1,192 were transplanted [8].

Despite the rapid developments of modern eye surgical techniques and instrumentations, corneal blindness remains a global public health issue [9] and this might be attributed to corneal patient's willingness to accept the corneal transplantation. In this regard, there was a scanty study regarding corneal transplantation acceptance. Hence, the main aim of this study was to determine the transplantation acceptance level and associated factors among corneal transplantation indicated patients at the University of Gondar Tertiary Eye Care and Training Center, Gondar, Ethiopia.

## 2. Method

### 2.1. Study Area, Design, and Period

University of Gondar Tertiary Eye Care and Training Center (UOGTECTC) is located in Gondar town, Northwest Ethiopia. It is 750 kilometers from Addis Ababa, the capital city of Ethiopia. The institute serves approximately 14 million people. The center provides eye care services to the Northwest part of the Ethiopian population. The University of Gondar Tertiary Eye Care and Training Center has two corneal specialists, three senior ophthalmologists, and 21 trainees from ophthalmic residents. This institution-based cross-sectional study was conducted from January 1, 2017, up to October 30, 2018.

### 2.2. Population

All corneal diseased patients with an age of beyond eighteen years visiting the UOGTECTC and having an indication for corneal transplantation were the source population. However, all corneal diseased patients with an age of beyond eighteen years visiting the UOGTECTC and willing for corneal transplantation from January 1, 2017, up to October 30, 2018, were the study population.

### 2.3. Inclusion and Exclusion Criteria

All corneal disease and transplantation indicated patients with an age of beyond eighteen years and willing to participate in the study were included, while those who are not volunteering to participate were excluded.

### 2.4. Sample Size and Sampling Technique

All beyond eighteen-year corneal patients who were willing for corneal transplantation and visited the UOGTECTC from January 1, 2017, up to October 30, 2018, were surveyed and sampled.

### 2.5. Operational Definition

  The blind: a corneal patient having a visual acuity level of less than 3/60 (snellen) in the better eye is blinded, whereas >6/18 as well as between 6/18 and 3/30 visual acuity levels were considered as normal and low vision, respectively [10].
  Corneal patient: a patient who is suffering from corneal disease-related complications.
  Good knowledge: a corneal patient was assessed for the knowledge level on corneal transplantation using seven knowledge assessing questions. A patient was considered as having good knowledge about corneal transplantation when he/she replied the correct knowledge, assessing questions above the mean score of the composite index. Otherwise, he/she was considered as having poor knowledge.
  Favorable attitude: a corneal patient was evaluated for his/her attitude towards corneal transplantation by five Likert-scaled questions and then dichotomized. A patient was considered as having a favorable attitude when he/she replied the correct attitude measuring questions above the mean score of the composite index. Otherwise, he/she was considered as having an unfavorable attitude.
  Corneal transplantation accepted: when a corneal disease and transplantation indicated the patient was willing to have surgery and sign the consent form.

### 2.6. Data Collection Procedure Quality Control

The data were collected at the corneal clinic of UOGTECTC using Amharic (the local and national language of Ethiopia) version interviewer-administered structured questionnaire. The questionnaire was first developed in English language and then back-translated to the Amharic language. The questionnaire had five components, such as sociodemographic, visual acuity status of the patient, knowledge, attitude, and indications as well as acceptance for corneal transplantation assessing questions. All the enumerators were the ophthalmology residents working in the corneal clinic. To assure the quality of the data and to make sure that all assessment team members were able to administer the questionnaires properly, a one-day rigorous training was given to the enumerators. Corneal diseased patients with an indication for CT identified by a senior corneal specialist were enrolled in the study after they signed the informed consent. Data collectors carried out role-play practices and then two-week pretest activities held in eight corneal patients who had an indication for corneal transplantation after being diagnosed by the senior ophthalmic specialists. The internal consistency (Cronbach's alpha) level of the pretest was done and found 0.96 for knowledge and 0.748 for attitude assessing questions, respectively. The questionnaire was adjusted accordingly to ensure the appropriateness and common understanding, and also the actual questionnaire was checked on a daily base by a primary investigator for completeness.

### 2.7. Data Processing and Analysis

Data were coded and entered into Epi-info version 7 and exported to SPSS version 20 software for analysis. Descriptive statistics were used to summarize characteristics of study participants and presented using text and tables. A simple logistic regression analysis was employed to assess the association between the exploratory variables and the acceptance level of corneal transplantation. The strength of the association was measured using the adjusted odds ratio (AOR) and 95% confidence interval (CI). A *p* value <0.05 was considered as a statistically significant predictor of corneal transplantation acceptance.

### 2.8. Ethical Consideration

Ethical clearance obtained from the University of Gondar College of Medicine and Health Science institutional review committee. The study participants were informed about the purpose of the research. The study participants were informed about their full right to participate or to refuse/withdraw the study at any time they want. A signed written consent was obtained from each study participant. The confidentiality of the information obtained was assured by coding and locking the data in a secure place.

## 3. Result

### 3.1. Sociodemographic Characteristics of the Respondents

A total of one hundred sixteen corneal patients having an indication for corneal transplantation have partaken. The mean age of the study participants was 51 (±21) years. The greater portion of the participants was found in the age of 51–70 years. A majority of the respondents (60.3%) were male. Almost all, 96.6% of the respondents were orthodox Christian. About fifty-five percent of the respondents were unable to read and write and were farmers. More than one-third (38.8%) of the respondents comes from very distant areas (Table 1).

### 3.2. Clinical Profile of Participants

Regarding clinical presentation, forty-two (28.2%) have low vision and forty-one (35%) have good vision. More than eighty-five percent of the participants had an optical indication for corneal transplantation (Table 2).

### 3.3. Knowledge and Attitude about Corneal Transplantation

The study revealed that about seventy-three percent of study participants had poor knowledge, while forty-seven percent of the respondents had an unfavorable attitude towards corneal transplantation (Table 3).

### 3.4. Acceptance Rate of Corneal Transplantation

The overall corneal transplantation acceptance level of the current study was only 38.8% (95% CI: 29.93, 47.66), in which 53.3% were male respondents. The greater rate, 44% of acceptance, was found in age of 18–32 years.

### 3.5. Reasons for Not Accepting the Corneal Transplantation

**Figure 1:**
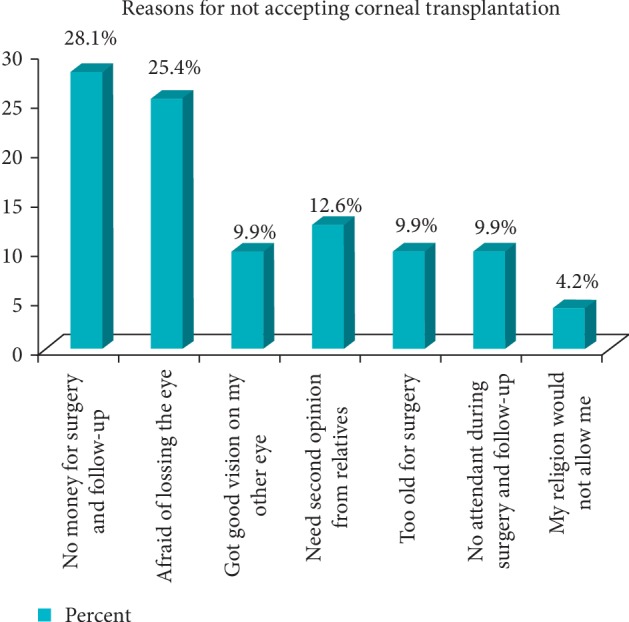
Reasons for the not accepting corneal transplantation in UOGTECTC, Northwest Ethiopia, 2018.

The main identified reason for not accepting corneal transplantation was “no money for surgery” followed by afraid of losing the eye (Figure 1).

### 3.6. Factors Associated with Acceptance of Corneal Transplantation

The result of binary logistic regression showed that knowledge and attitude were significantly associated with acceptance of CT. As a result, those study participants who had poor knowledge were 2.41 times [AOR = 2.41; 95% CI: 1.90, 6.48] more likely not to accept CT as compared to those who had good knowledge. Those participants who had an unfavorable attitude towards CT were 6.3 times [AOR = 6.33; 95% CI: 2.42, 16.54] more likely not to accept CT as compared to those with a favorable attitude (Table 4).

## 4. Discussion

In this study, about thirty-nine percent of corneal transplantation indicated patients had accepted the transplantation. This result is higher than the study finding of cataract surgery in Tanzania (17.8%) [11]. The possible justification for this discrepancy could be the difference in the study design; the current study was cross-sectional, while the study in Tanzania was cohort. Similarly, some patients in the current study might sign the consent for the surgery without getting the transplantation service. This might increase the acceptance of corneal transplantation.

Regarding barriers to acceptance of CT surgery, this study found out inability to afford for the surgery service accounts for 22 (28.2%). This result is comparable with studies done in Nigeria [12], for a barrier of ophthalmic surgery found out that 28.3% barrier is the cost of the hospital service. Our study finding is smaller than a study done in the outreach program of central Ethiopia (91.8%) [13]. This might be due to the fact the current study was done in the hospital, whereas most patients do not come mostly due to economic reasons, while the latter was at the outreach areas, which might create discrepancies in figures.

The second barrier found in the study is afraid of losing vision, which was found in 18 (25.4%) patients. This is comparable to a study conducted in India [14]. Barriers to the uptake of cataract surgery in patients presenting to a hospital were fear of surgery, which accounts for 34%.

Got good vision in my other eye is another barrier identified in this study, and it accounts for 7 (9.9%). Compared to barriers to cataract surgery done in the outreach program of central Ethiopia [13] where good vision in the fellow eye accounts for 39.7%. This might be again because the study was done on the outreach program, whereas our study was done in a hospital where patients come seeking treatment for their diseased eye.

About 7 (9.9%) of barriers are accounted by not having an assistant during surgery as well as during follow-ups. This result is lower than the study finding on barriers of trichiasis surgery conducted in the Amhara region, 31% [15]. This discrepancy might be even though the social and cultural makeup is similar; the study is population based and has a large sample size.

In this study, sixty-six of the respondents were male and did not accept CT, while 54.3% were females, which seems like females are more likely to accept CT. But the study was conducted in India [14] based on barriers of cataract surgery considering female gender as a barrier to cataract surgery and accounting for 27%. This might be due to the social and cultural difference between the study areas.

The age group between 18 and 32 years has found to accept CT surgery (45.7%) than other older age groups. This result is consistent with the cohort study on acceptance of cataract surgery in Tanzania [11] where they found out older patients are less likely to accept surgery than younger. In this study, patients who are blind (71.4%) did not accept CT. This is also consistent with the result obtained from Tanzania [11].

The study also identified the factors which influence the corneal transplantation acceptance. The first root cause was knowledge. It is found that patients with poor knowledge were 2.41 more likely to not accept CT than patients with good knowledge (CI: 1.90, 6.48). This could be due to lack of awareness of the communities about the eye health and care, particularly corneal transplantation.

This study shows a high association between attitude and tendency to accept CT. It has been found that patients with an unfavorable attitude were 6.33 times more likely not to accept CT as compared to those with a favorable attitude (CI 2.42, 16.54). This is consistent with the study conducted in India, where attitudinal barriers are more reported than accessibility barriers. Similarly, the study finding of Tanzania even with a ‘bridging strategy accessible and affordable,' the acceptance of surgery is low.

## 5. Limitations of the Study

Due to the cross-sectional nature of the study, it is difficult to distinguish whether the cause preceded the effect. And the other limitation was too difficult to obtain published articles in the current study discipline.

## 6. Conclusion

In this study, a large proportion of corneal transplantation indicated patients did not accept corneal transplantation surgery. Poor knowledge and an unfavorable attitude towards corneal transplantation surgery were found to be statistically associated with CT acceptance.

## Figures and Tables

**Table 1:** Sociodemographic characteristics of the respondents in UOGTECTC, Northwest Ethiopia, 2018 (*n*=116).

Variable	Category	Frequency	Percent
Age (years)	18–32	29	25
	33–50	31	26.7
	51–70	37	31.9
	71–98	19	16.4
Sex	Male	70	60.3
	Female	46	39.7
Religion	Orthodox	112	96.6
	Muslim	4	3.4
Residence	Urban	29	25
	Rural	87	75
Educational status	Unable to read or write	76	65.5
	Primary	30	25.9
	Secondary and above	10	8.6
Occupation	Farmer	75	64.6
	Governmental employee	4	3.4
	Merchant	6	5.2
	Others^*∗*^	31	26.8
Monthly income	<500	34	29.3
	500–999	40	34.5
	1000–2000	26	22.4
	≥2001	16	13.8
Distance from UOGTECTC (in km)	Less than 175	71	61.2
	More than 175	45	38.8
Money spent for transportation	<68.4 birr	71	61.2
	≥68.4 birr	45	38.8

^*∗*^Student, daily laborer, and housewife.

**Table 2:** Clinical profile of patients visiting UOGTECTC in Gondar town, Northwest Ethiopia, 2018 (*n*=116).

Variable	Category	Frequency	Percent
Vision status	Blind	42	36.2
	Low vision	33	28.4
	Good vision	41	35.3
Indication for CT	Optical purpose	102	87.9
	Others^*∗*^	14	12.1

^*∗*^Cosmetics, therapeutic, and reconstructive.

**Table 3:** Knowledge and attitude about corneal transplantation among patients visiting UOGTECTC in Gondar town, Northwest Ethiopia, 2018 (*n*=116).

Variable	Category	Frequency	Percent
Knowledge	Good knowledge	31	26.7
	Poor knowledge	85	73.3
Attitude	Favorable attitude	61	52.6
	Unfavorable attitude	55	47.4

**Table 4:** Factors associated with CT acceptance in UOGTECTC, Northwest Ethiopia, 2018.

CT accepted					
Variable	Yes	No	COR (95% CI)	AOR (95% CI)	*p* value
Residence					
Rural	27	60	3.63 (1.51, 8.74)	3.09 (0.14, 2.40)	0.87
Urban	18	11	1.00	1.00	

Sex					
Male	24	46	1.61 (0.75, 3.45)		
Female	21	25	1.00		

Knowledge level					
Good	19	12	1.00		
Poor	26	59	3.59 (1.52, 8.47)	2.41 (1.90, 6.48)	0.023

Attitude level					
Favorable	37	24	1.00		
Unfavorable	8	47	9.06 (3.65, 22.47)	6.33 (2.42, 16.54)	0.001

Vision status					
Blind	12	30	1.77 (0.71, 4.41)		
Low vision	16	17	0.75 (0.29, 1.89)		
Good vision	17	24	1.00		

Money spent for transportation					
<68.4 birr	24	47	1.71 (0.79, 3.68)		
≥68.4 birr	21	24	1.00		

## Data Availability

The data can be accessed from the corresponding author through getasewmulat@gmail.com with reasonable requests.

## Abbreviations

AOR: Adjusted odds ratio
CI: Confidence interval
COR: Crude odds ratio
CSA: Central statistical agency
CT: Corneal transplantation
Epi-Info: Epidemiological information
SPSS: Statistical package for social scientists
UOGTECTC: University of Gondar tertiary eye care and training center.

## Supplementary Materials

Supplementary Materials Knowledge and attitude assessing characteristics are given in the supplementary information file.
