# Supplementary material for: Are Corneal Patients Accepting the Transplantation? The Cases of University of Gondar, Tertiary Eye Care and Training Center, Ethiopia
Source: J Ophthalmol. 2019 Dec 13;2019:4560649. doi: 10.1155/2019/4560649 (PMC6930795; doi:10.1155/2019/4560649)
Supplement: Supplementary Materials — Knowledge and attitude assessing characteristics are given in the supplementary information file. [file 4560649.f1.docx]

**ANNEX**

Knowledge assessing characteristics

| **S.N** | **Variable** | **Category** | **Skip pattern** |
| --- | --- | --- | --- |
| Q301 | Have you ever heard about corneal transplantation? | 1. Yes 2. No | If no ->Q303 |
| Q302 | If yes, where do you hear it? | 1. Neighbor 2. TV/Radio/Social Media 3. People who had the procedure 4. Other (specify)__________ |  |
| Q303 | What is corneal transplantation? | 1. It is removing the whole eye and replacing it with new one 2. Its only removing the diseased corneal and replacing it with a new one 3. It’s removing the diseased cornea and leave it 4. Other (specify)… |  |
| Q304 | Where do you think the donor cornea come from? | 1. From animals’ eye 2. Synthetic corneas manufactured in laboratory 3. From a living family member 4. Other (specify)……. |  |
| Q305 | Who do you think will perform the operation? | 1. All physician can perform the operation 2. All eye doctors can perform the procedure 3. Only eye specialist doctor who is trained on this specific Procedure 4. Eye nurses who are trained on this specific procedure 5. Eye nurses who are trained on this specific procedure |  |
| Q306 | What is the benefit of corneal transplantation? | ………………………..……… |  |
| Q307 | Did you know corneal transplantation is done in UOGH tertiary eye care and training center? | 1. Yes 2. No |  |
| Q308 | For how long should a corneal transplant patient follow, after the surgery? | 1. for only one year 2. for only two year 3. Life long 4. Other (specify) |  |
| Q309 | How much do you think corneal transplantation cost in UOGH? (Both for surgery and follow up) | 1. It is free 2. less than 2000 birr 3. More than 2000 Birr 4. Other (specify) |  |

**Attitude assessing characteristics**

| **S.N** | **Variable** | **Strongly Disagree** | **Disagree** | **Neutral** | **Agree** | **Strongly Agree** |
| --- | --- | --- | --- | --- | --- | --- |
| Q401 | I feel CT can improve my vision |  |  |  |  |  |
| Q402 | I have a fear of having eye surgery |  |  |  |  |  |
| Q403 | I believe UOGH corneal sub specialists are well trained and can perform CT |  |  |  |  |  |
| Q404 | I feel CT may not help improve my quality of life since I can manage my daily work. |  |  |  |  |  |
| Q405 | Since I have good vision on the other eye, I think I don’t need CT. |  |  |  |  |  |
